# S216. REDUCED EMOTIONAL FACIAL EXPRESSION IN SCHIZOPHRENIA – A PROBE INTO THE PHENOMENOLOGY AND RELEVANCE OF EXPRESSIVE NEGATIVE SYMPTOMS

**DOI:** 10.1093/schbul/sby018.1003

**Published:** 2018-04-01

**Authors:** Marcel Riehle, Stephanie Mehl, Tania M Lincoln

**Affiliations:** 1 University of Hamburg; 2 Frankfurt University of Applied Sciences

## Abstract

**Background:**

Emotional facial expressions are vital communicative signals and a lack thereof should interfere with successful social interaction. That people with schizophrenia lack emotional facial expression, mostly irrespective of antipsychotic medication, is not only a well-known notion but also backed up by ample evidence. However, a closer look reveals a more complicated picture that implies that maybe only those with expressive negative symptoms (ENS; i.e. blunted affect and alogia) but not those without ENS show reduced facial expressiveness. Furthermore, while the reduction has been found consistently for positive facial expressions, the evidence for negative facial expressions has been mixed. Finally, the social consequences of the reduction are mostly unknown and thus whether or not the reduction actually interferes with social interactions. To address these questions, we tested for the symptom-specificity of reduced positive and negative facial expression (phenomenology) and their social relevance in patients with schizophrenia with versus without ENS.

**Methods:**

The frequency of positive and negative facial expressions in an affiliative role-play were assessed with the Facial Expression Coding System (FACES) in people with schizophrenia with (n = 18) and without ENS (n = 30) and in healthy controls (n = 39). Based on observing the role-play, independent raters also rated their willingness for future interactions with each participant. The presence of ENS was assessed via the Positive and Negative Syndrome Scale (PANSS).

**Results:**

Patients with schizophrenia and ENS did not differ on positive symptoms and depression or on chlorpromazine equivalent medication dosage from those without ENS. The analysis of the frequency of facial expressions revealed that patients with ENS showed reduced levels of positive facial expressions both compared to those without ENS (d = -0.82) and to controls (d = -1.21). Both patient groups (with and without ENS) showed equally reduced negative facial expressions compared to controls (ds = -0.99 and -0.86). Raters also indicated less willingness for future interactions with patients with ENS than without ENS (d = -0.92). This difference was significantly mediated by the reduced positive facial expressions.

**Discussion:**

The findings offer new insights into the phenomenology and the relevance of reduced emotional facial expression in schizophrenia. Our study indicates that the moderate to large mean differences that have been reported in earlier studies comparing samples with more broadly defined schizophrenia to healthy controls could mainly be driven by a reduction in facial expressions that is relatively specific to those with ENS. However, some aspects of reduced facial expression may nevertheless be genuine to more broadly defined schizophrenia given that we found patients with schizophrenia both with and without ENS to exhibit reduced levels of negative facial expressions. Finally, we found that the reduction of the positive facial expressions explained why raters were more willing to interact with those without ENS than with those with ENS. This further highlights the relevance of ENS by showing that they interfere with successful social interaction and go along with immediate social costs.

